# Importance of CBCT setup verification for optical‐guided frameless radiosurgery

**DOI:** 10.1120/jacmp.v15i3.4487

**Published:** 2014-05-08

**Authors:** Lei Fu, Harold Perera, Xiao Ying, Yan Yu

**Affiliations:** ^1^ Radiation Oncology Department Thomas Jefferson University Philadelphia PA USA

**Keywords:** stereotactic radiosurgery, optical guidance, cone‐beam CT verification, frameless localization, patient setup

## Abstract

The purpose of this study is to quantify the discrepancy between optical guidance platform (OGP) frameless localization system (Varian) and Trilogy on‐board imaging (OBI) system (Varian) for setting up phantom and stereotactic radiosurgery (SRS) patient; and to determine whether cone‐beam CT (CBCT) is necessary for OGP patient setup, and compare CBCT and orthogonal kV‐kV in term of their verification capability. Three different phantoms were used in the study: a custom‐made phantom, a Penta‐Guide phantom, and a RANDO phantom. Five patients using both OGP and CBCT setup and 14 patients using CBCT setup alone were analyzed. One patient who had big couch shifts discrepancy between OGP and CBCT was selected for further investigation. Same patient's CBCT and planning CT were fused. A RANDO phantom simulation experiment was performed using OGP setup with both CBCT and orthogonal kV‐kV verification. For all of three phantom experiments, the shifts performed by CBCT beam and orthogonal kV–kV were all within 1 mm. Among five SRS patients using OGP setup, three had 3D couch corrections more than 3 mm. The image fusion of CBCT and planning CT clearly illustrated a tilt of bite‐block in a patient's mouth. For 14 SRS patients using CBCT‐guided setup, overall 3D correction was 3.3 ± 1.5 mm. RANDO phantom experiment demonstrated how a tilted bite‐block caused isocenter shift. CBCT‐calculated shifts are the same as expected, but kV–kV results differed by 1–2 mm if the initial head position is tilted. The bite‐block tilting in patient's mouth is a major reason for the cause of positioning error for OGP frameless SRS setup. CBCT verification is necessary. CBCT provides more accurate couch corrections than orthogonal kV–kV when head was tilted. OGP is useful for detecting patient movement, but it does not necessarily imply that the isocenter has moved.

PACS numbers: 87.55.km, 87.55.Qr, 87.53.Ly

## I. INTRODUCTION

Since Leksell first introduced “radiosurgery” in 1951, many commercial systems have been developed to deliver accurate stereotactic radiosurgery (SRS) plans. While some of these systems still use invasive frame‐based localization device, now they also permit frameless localization and positioning, including completely IGRT‐guided system such as the CyberKnife system (Accuray, Sunnyvale, CA) and Novalis radiosurgery system (BrainLAB AG, Feldkirchen, Germany). Optical guidance platform (OGP) (Varian Medical Systems, Palo Alto, CA) is one of the systems for intracranial stereotactic radiosurgery (SRS) target localization and patient positioning. It can be used with either frame or frameless array. The frameless array is a noninvasive positioning system that can monitor the patient's positioning in real time. In this system, the patient's head is immobilized with a mask while the positioning is tracked through the use of a bite‐block, which is not attached to the mask but to patient's maxillary dentition. Room‐mounted infrared video cameras determine the position and orientation of the bite‐block.^(1)^ The treatment outcomes using frameless localization have been reported to compare favorably to the frame‐based system.^(2)^

Before kilovoltage (kV) imaging and cone‐beam computed tomography (CBCT) became available on the treatment machine, the overall positioning accuracy of optical guidance frame‐less system was not easy to obtain. It includes, for example, camera calibration error, patient repositioning error during both CT scan and treatment, CT scanner error, and error from treatment planning software when identifying the infrared reflective ball position. The localization accuracy on phantom is 1.11±0.3mm based on University of Florida's early study.^(3)^ The authors assumed that the maxillary dentition is a rigid structure accessible for head position determination^(1)^ Later MV portal film was used for verification and 3 mm displacement has been observed.^(4)^ The University of California, San Diego (UCSD) has used kV‐kV to verify positioning and reposition the patients if isocenter placements were greater than 2 mm.^(5)^ One study assessed OGP frameless system using CBCT on both Elekta and Varian linacs and found maximum 4.4 mm difference.^(6)^

In this paper, optical‐guidance frameless system positioning accuracy using imaging verification methods was evaluated on both phantoms and patients. First we examined the setup difference between OGP and CBCT, and between OGP and kV–kV on the phantom. Then, we examined the couch difference between OGP and CBCT on patients. One patient case has been further investigated by performing a RANDO phantom simulation experiment. Finally, the feasibility of using CBCT alone for SRS setup was discussed.

## II. MATERIALS AND METHODS

### A. System and phantom description

In our clinic, we use Trilogy linear accelerator (Varian Medical Systems), FastPlan treatment planning software (Varian Medical Systems), Pinnacle treatment planning software (Philips Healthcare, Andover, MA), and MOSAIQ R&V software (Elekta, Stockholm, Sweden). Phantom tests were performed on three different phantoms: an in‐house plexicube phantom, Penta‐Guide phantom (Modus Medical Devices Inc., London, Canada), and RANDO phantom (The Phantom Laboratory, Salem, NY).

**Figure 1:**
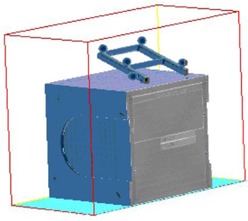
Plexicube phantom with bite tray attached.

The plexicube phantom is shown in Fig. 1. We modified it by fixing a bite‐block on one of the center slabs. There are markers on the anterior and lateral sides for laser alignment and three 7 mm diameter metal balls in the center slab for imaging verification.

**Figure 2:**
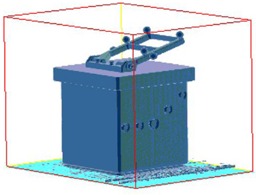
Penta‐Guide phantom with bite tray attached.

The Penta‐Guide phantom was used for daily IGRT QA in our clinic (Fig. 2). It has an accessory to attach the bite‐block on top of the phantom.

**Figure 3:**
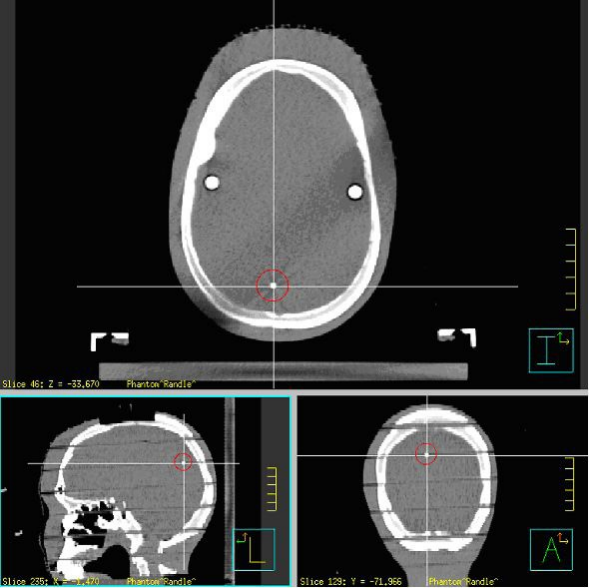
RANDO phantom with BBs.

**Figure 4:**
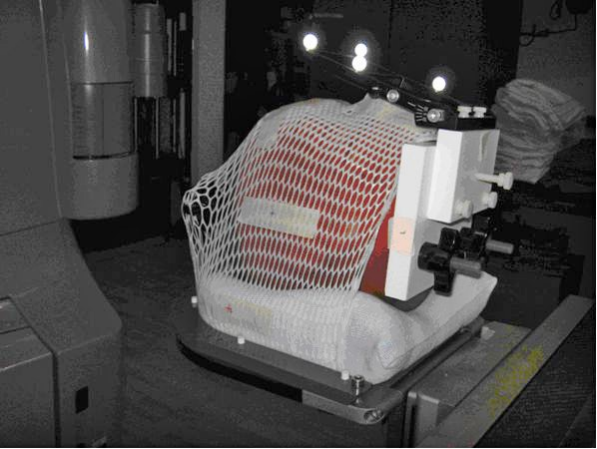
RANDO phantom with bite‐block is immobilized on SRS table end attachment.

We put two BBs inside the RANDO head phantom at two different locations (Fig. 3). The bite‐block was fixed on a small block, which was connected with another large block by screws. The large block was fixed on the inferior surface of the RANDO phantom. The small block can be adjusted to tilt in small angles by a screw in the center of the block (see Fig. 4).

### B. Phantom experiments

Isocenter localization and patient positioning on Varian Trilogy was implemented by either a six degrees of freedom (DOF) couch or a regular couch with a three DOF couch mount attached at the couch end, which can be adjusted up and down, tilt and spin. In our clinic, we use a regular couch and the couch mount. The OGP system includes two infrared cameras and a bite‐block with six reflective balls. Bite‐block is fitted in patient's mouth before simulation and ten reseating tests must be done to make sure bite‐block position in the mouth is reproducible. The tolerance of average reseating error is 0.75 mm.

The experiments on three phantoms were performed to assess the setup differences between OGP, kV‐kV, and CBCT. The processes are the same for three phantoms. First, we CT scanned the phantoms with 2 mm slice thickness and exported the images to Pinnacle and FastPlan treatment planning systems, where the same isocenter was identified on both systems. Then reference images were exported to MOSAIQ and optical guidance system, respectively. Next, on Trilogy, OGP was used to position the phantom. kV–kV and CBCT images were acquired and registered with DRRs or reference CT images to obtain the couch corrections. The couch corrections include x, y, z translation and couch rotation. Our monthly OBI quality assurance test assures that the kV, CBCT, and MV source isocenter coincidence is within 1 mm.

### C. Patient study

Starting in June 2008, 21 patients received single‐fraction brain SRS treatment, and two patients received multifraction SRT treatments. The first four patients were treated using only optical‐guidance system for positioning. From April 2009 to June 2010, five SRS patients were set up by OGP system and verified by either CBCT or kV–kV, or both, before treatment. All of the patients passed the reseating test successfully before CT scan. A treatment plan was created by FastPlan cone‐based arc planning technique. The patient's position was adjusted if the couch shifts calculated by CBCT is greater than 2 mm. From 2011 to present, 14 patients were set up by laser first and then repositioned by using CBCT. They included two multifraction SRT patients. Treatment plan was created by Pinnacle conformal beams. All patients were immobilized by thermoplastic mask. The couch corrections were recorded by therapists for each treatment in MOSAIQ.

Among the patients initially positioned by OGP, we chose one patient whose couch shift calculated from CBCT is in disagreement with OGP setup by 7 mm. This patient's CBCT and planning CT were imported into MIM 6.7 (MIM Software Inc., Cleveland, OH) for fusion and registration. The fused images showed that the bite‐block was in a different position in patient's mouth. There was about a 3° tilt difference in bite‐block position between CBCT and planning CT. For this patient, we repositioned the bite‐block twice before treatment, and CBCT found the same couch corrections. This discrepancy might have been caused by incorrect biting position during CT scan. Although this patient passed the reseating test before CT simulation in a sitting position, it is still possible that the bite‐block position could have been changed due to the lying position at the simulator for a long time, especially for the patients who have fewer or no teeth, or patients who cannot hold the bite‐block in place for a long time.

### D. RANDO phantom study

**Figure 5:**
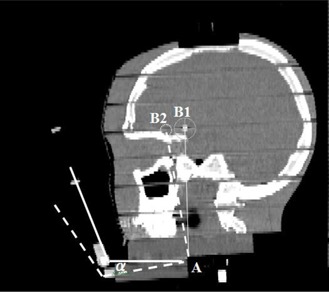
The sagittal view of a RANDO phantom. Point A is the rotation axis, point B1 is the isocenter. The dashed line shows the bite‐block and isocenter position after rotation around A.

A RANDO phantom study was performed to investigate how the bite‐block displacement in the mouth would affect the final positioning, as well as to compare CBCT with kV–kV regarding their capability for setup verification. As seen in Figs. 4 and 5, the bite‐block was fixed on a RANDO phantom through two other blocks in a way that the angle of one block can be changed. It mimicked the situation that the bite‐block angulated in patient's mouth. After initial setup using OGP, bite‐block was tilted from 0.5° to 3° in intervals of 0.5°. Since there was no rotation along the longitudinal direction to the bite‐block, there was no lateral shift. Also due to the particular location of the BB, the longitudinal shift was very small. The couch vertical correction on the OGP screen was recorded as the expected couch correction. For each angle change to the bite‐block, couch was adjusted to the “planned” position by making all translational and rotational offsets to zero on OGP computer screen. Then, both CBCT and kV–kV images were acquired and couch corrections from CBCT and kV‐kV were compared to the expected couch corrections, respectively.

## III. RESULTS

For both plexicube and Panta‐Guide phantoms, the shifts calculated by cone beam, kV, and MV were all within 1 mm, after positioning the phantom using optical guidance system. Table 1 provides the patients' couch corrections calculated by CBCT after OGP setup. Among five patients, three had 3D couch correction of 6 mm or more, two had less than 3 mm. The disagreement between CBCT and OGP setup for patients is significantly more than the phantoms. However, due to the small number of patients treated using OGP setup in our clinic, statistical significance cannot be generalized from these data. For patients whose couch corrections were greater than 3 mm, we repositioned the patients based on CBCT.

**Table 1:** Patient CBCT verification with OGP setup

	*Couch Shift (mm)*			
*Patient Number*	*Vertical*	*Longitudinal*	*Lateral*	*3D*
1	−7	6	−4	10
2	3	6	0	6.7
3	0	2	2	2.8
4	0	−2	1	2.5
5	6	−2	2	6.6

**Figure 6:**
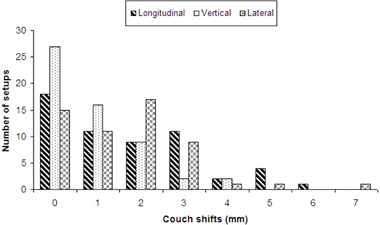
Number of setups for each shift group from 0 to 7 mm in three couch directions.

For 14 patients positioned by using CBCT only, 12 SRS patients had average 3D couch correction of 2.4±1.8mm; two SRT patients had total 45 CBCT shifts recorded and their average 3D couch corrections were 3.8±1.5mm and 3.1±1.4mm, respectively. The overall 3D correction for all 56 CBCT is therefore 3.3±1.5mm. This result is similar to the work by Masi et al.^(7)^ and Peng et al.^(6)^ (3.2±1.5mm), but their data are on Elekta XVI. Meanwhile, as illustrated in Fig. 6, the numbers of setups with no vertical shift were significantly higher than with the other two directions. Vertical shifts greater than 3 mm were rare. The numbers of setups of having 0, 1, 2, and 3 mm shift in longitudinal and lateral direction were about the same.

**Figure 7:**
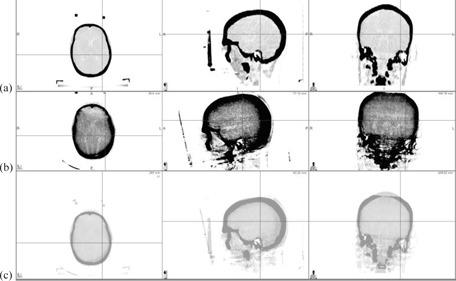
Patient's (a) planning CT, (b) cone‐beam CT, and (c) fusion results.

The fusion of one patient's planning CT with CBCT is shown in Fig. 7. It clearly shows that the bite‐block tilted in the mouth. Table 2 shows that for this particular phantom, 1° tilt can cause 2 mm discrepancy between OGP and CBCT.

**Table 2:** Comparison of CBCT and kV calculated shifts with expected shifts

*Bite‐block Tilt Angle (°)*	*Expected Couch Vertical Correction (mm)*	*Couch Vertical Correction by kV‐kV (mm)*	*Couch Vertical Correction by CBCT (mm)*	*Deviation Between kV‐kV and CBCT (mm)*
0.5	1	1	1	0
1	2	3	2	1
1.5	3	4	3	1
2	4	5	4	1
3	6	8	6	2

**Figure 8:**
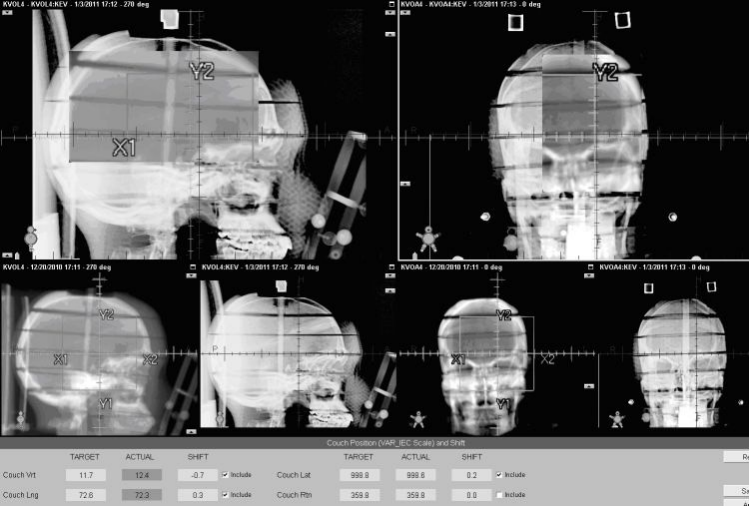
KV image of 3° tilt phantom overlayed with DRR.

**Figure 9:**
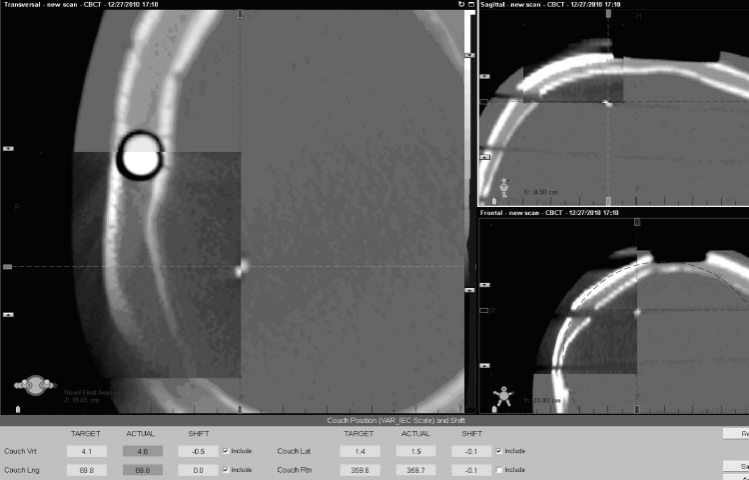
CBCT images at 2.5° tilt. Images inside the spyglass are planning CT

Table 2 also compares kV and CBCT couch vertical correction with expected shifts. CBCT results are equal to the expected, but kV results showed a discrepancy of 1−2 mm. The error became larger when tilt increased. Figure 8 shows the kV images of RANDO phantom at 3° tilt. The tilted bony structure became difficult to align with DRR. Figure 9 shows the CBCT images of RANDO phantom at 2.5° tilt. Although bony structures are aligned well, the actual isocenter is off about 1 mm.

## IV. DISCUSSION

Patient's lower jaw is not a rigid body. Biting the tray sometimes is difficult for older patients without teeth or for very sick patients. Table 2 shows that the angle change of bite‐block as small as 1° could cause the isocenter to be shifted by 2 mm or more, depending on where the isocenter is located. It implies that if the patient cannot hold the bite‐block in the mouth at the same position during either CT scan or localization, a significant setup error may occur. In the earlier design of noninvasive stereotactic frames, upper dentition is used to achieve relocation, “the position is maintained by means of a series of straps which pass over the head”;^(8)^ and “once the patient is in position, the patient has full movement of the lower jaw and can speak freely”.^(9)^ In later frameless bite‐block systems, it is “attached to the upper dentition or gums by having the person bite down”.^(1)^ Using tape around patient's jaw to help the patient hold the bite‐block may be useful, but is not sufficient. The optical guidance is a valuable tool for realtime monitoring of the motion, but bite‐block alone without any additional support presents a potential problem that it may move due to the patient's jaw movements, even when there are no head movements. In that situation, it should not be considered as target shift and should not be corrected. Reimaging is necessary if the motion still exists after having the patient bite the block tightly.

Thus it is essential to use other methods, such as kV‐kV imaging or CBCT, to verify OGP setup accuracy. Our study shows that the CBCT‐calculated shifts are more accurate than kV–kV shifts. CBCT‐calculated shifts are the same as expected shifts, but kV–kV differs by 1–2 mm if the initial head position is tilted. This is not surprising because CBCT images contain more information for fusion than kV–kV; it is more difficult for kV–kV to generate better matching between a reference image and a tilted image.

However, using CBCT for setup verification has some limitations. It takes longer time and delivers more radiation dose to patients than kV–kV. It requires couch lateral and rotation to be at or close to zero to avoid gantry and couch collision, thus it can't be used for per‐beam positioning verification that can be performed by OGP or other radiosurgery systems. The CBCT positioning accuracy is limited by such issues as the CBCT isocenter vs. radiation isocenter calibration, pixel size resolution, and four degrees of freedom couch correction approximation.

If the bite‐block is tilted, the patient's head will be tilted and cannot be corrected completely by translations. Figure 9 is a good example to demonstrate this problem. For our particular phantom, the BB was off about 1 mm for head tilting of 2.5° even if the bones matched well. A 6D couch can solve this problem; otherwise bigger PTV margin is needed for planning.

## V. CONCLUSIONS

The discrepancy between CBCT and OGP for setting up phantoms was less than 1 mm, but may be greater for setting up SRS patients. The bite‐block tilting in patient's mouth is a major reason for the cause of this discrepancy. CBCT provides more accurate couch corrections than orthogonal kV‐kV for SRS frameless patient setup verification when head was tilted. OGP is useful for monitoring patient movement, but it does not necessarily imply that the isocenter has moved.
